# Metabolomic Profiling of Tongue Coating Reveals Potential Molecular Features Linked to Type 2 Diabetes Progression

**DOI:** 10.3390/ijms27083375

**Published:** 2026-04-09

**Authors:** Po-Chi Hsu, Pei-Yung Liao, Tse-Yen Yang, Hen-Hong Chang, John Y. Chiang, Yu-Chuen Huang, Lun-Chien Lo, Der-Yen Lee

**Affiliations:** 1 School of Chinese Medicine, China Medical University, Taichung 404328, Taiwan; bryanhsu0813@gmail.com (P.-C.H.); yuchuen@mail.cmu.edu.tw (Y.-C.H.); 2 Department of Chinese Medicine, China Medical University Hospital, Taichung 404332, Taiwan; tcmchh55@gmail.com; 3 Graduate Institute of Chinese Medicine, China Medical University, Taichung 404328, Taiwan; lpy5433@gmail.com; 4 Department of Internal Medicine, Division of Endocrinology and Metabolism, Changhua Christian Hospital, Changhua 50006, Taiwan; 5 Molecular and Genomic Epidemiology Center, Department of Medical Research, China Medical University Hospital, Taichung 404332, Taiwan; hardawayoung@gmail.com; 6 Department of Medical Laboratory Science and Biotechnology, College of Medical and Health Science, Asia University, Taichung 41354, Taiwan; 7 Center for General Education, China Medical University, Taichung 404328, Taiwan; 8 Master Program of Digital Health Innovation, College of Humanities and Sciences, China Medical University, Taichung 404328, Taiwan; 9 Graduate Institute of Integrated Medicine, China Medical University, Taichung 404328, Taiwan; 10 Chinese Medicine Research Center, China Medical University, Taichung 404328, Taiwan; 11 Department of Computer Science and Engineering, National Sun Yat-Sen University, Kaohsiung 80424, Taiwan; chiang@cse.nsysu.edu.tw; 12 Department of Healthcare Administration and Medical Informatics, Kaohsiung Medical University, Kaohsiung 80708, Taiwan; 13 Department of Medical Research, China Medical University Hospital, Taichung 404332, Taiwan

**Keywords:** metabolomics, diabetes diagnosis, tongue coating, biomarkers, diabetes-associated dysregulation, mass spectrometry

## Abstract

Diagnosis and monitoring of type 2 diabetes mellitus (T2DM) typically rely on invasive blood-based biomarkers. To explore non-invasive alternatives, this study examined tongue coating metabolites to identify metabolic signatures linked to diabetes progression. A case-control observational study categorized participants into control, prediabetes, and diabetes groups. Tongue coating samples were analyzed using liquid chromatography-mass spectrometry (LC-MS). Differential metabolites were correlated with clinical parameters, including HbA1c, BMI, and eGFR. Distinct metabolic profiles emerged across groups, with significant differences in five endogenous metabolites (phenylpyruvic acid, propionylcarnitine, pyridoxal 5′-phosphate, phenethylamine, phenethylamine glucuronide) and four amino acids (isoleucine, lysine, phenylalanine, tyrosine). Diabetic subjects showed elevated phenylpyruvic acid and phenethylamine, while propionylcarnitine, pyridoxal 5′-phosphate, and phenethylamine glucuronide were reduced. Phenethylamine was positively correlated with HbA1c; propionylcarnitine and phenethylamine glucuronide showed negative correlations with HbA1c and BMI. Detected total amino acids were inversely correlated with eGFR. Additionally, a diabetes index derived from these metabolic features also holds potential for discriminating disease states. These findings underscore the potential of tongue coating metabolites as a relatively non-invasive approach for evaluating T2DM states. The observed metabolic alterations provide valuable insights into diabetes-associated dysregulation, including protein glycation, obesity-related metabolic shifts, and renal impairment.

## 1. Introduction

Type 2 diabetes mellitus (T2DM) is a chronic metabolic disorder characterized by persistent hyperglycemia, insulin resistance, and β-cell dysfunction. It is a leading cause of morbidity and mortality worldwide, contributing to cardiovascular disease, nephropathy, neuropathy, and retinopathy [1,2]. Despite extensive research on diabetes biomarkers, most studies rely on blood, urine, or saliva, while alternative biofluid sources remain underexplored. Currently, metabolomics has been applied in the analysis of new biomarkers for T2DM and its complications, such as branched-chain amino acids, phenylalanine metabolites, and metabolites involved in energy and lipid metabolism [3,4,5]. However, these metabolomics studies predominantly rely on invasive sample collection methods involving serum and plasma [6,7].

Diabetes has systemic impacts affecting virtually all tissues, including significant oral manifestations. Accumulating evidence indicates that oral health issues, including periodontitis, oral microbiota dysbiosis, and altered salivary metabolites, are prevalent among diabetic patients. Furthermore, oral complications correlate strongly with an increased incidence of diabetes-related microvascular and macrovascular diseases [8,9,10]. Thus, oral tissues and biofilms, such as tongue coating, represent an innovative, yet largely untapped resource for non-invasive diabetes biomarker discovery.

Tongue coating, a biofilm composed of exfoliated epithelial cells, oral microbiota, and various metabolites, presents distinct advantages as a diagnostic matrix owing to its non-invasive accessibility, ease of collection, and potential to mirror systemic metabolic states. Recent studies have begun exploring the metabolomics of tongue coating in various diseases, demonstrating distinct metabolic patterns associated with precancerous lesions of the stomach and chronic hepatitis. In patients with gastric precancerous lesions presenting phlegm-dampness syndrome, tongue-coating metabolites exhibit abnormalities in lipids and lipid-like molecules, particularly in glycerophospholipid metabolism. In addition, the urine of patients with chronic hepatitis demonstrates correlations between energy, amino acid, and nucleotide metabolism and the type of tongue coating [11,12]. These preliminary findings strongly suggest the potential utility of tongue coating metabolomics for disease-specific biomarker identification.

Nevertheless, to our knowledge, no prior study has characterized the metabolomic signature of tongue coating in individuals with diabetes mellitus. Addressing this critical research gap, our study aims to perform comprehensive metabolomic profiling of tongue coating samples from diabetic and prediabetic individuals to identify metabolic signatures associated with T2DM. Additionally, by examining correlations between these putatively identified metabolites and key clinical parameters, including glycated hemoglobin (HbA1c), body mass index (BMI), and estimated glomerular filtration rate (eGFR), we aim to explore their potential in clinical diabetes management. Our findings could pave the way for non-invasive, metabolomics-based diagnostic approaches.

## 2. Results

### 2.1. Demographic and Clinical Characteristics of Participants

The demographic and clinical characteristics of participants across control (*n* = 26), prediabetes (*n* = 14), and type 2 diabetes mellitus (T2DM, *n* = 120) groups are summarized in Table 1. Participants in the T2DM group were significantly older (64.89 ± 9.78 years) compared to the control group (53.96 ± 21.42 years; *p* < 0.001). Body mass index (BMI) was significantly higher in diabetic subjects (27.53 ± 4.71 kg/m^2^) compared to the control and prediabetes groups (*p* < 0.001). Glycemic control, indicated by glycated hemoglobin (HbA1c), was notably poorer in T2DM patients (6.96 ± 0.85%) compared to prediabetic participants (5.88 ± 0.42%; *p* < 0.001). Additionally, diabetic subjects exhibited significantly elevated fasting blood glucose levels (133.95 ± 36.60 mg/dL vs. 111.00 ± 13.21 mg/dL in prediabetes; *p* = 0.022). Estimated glomerular filtration rate (eGFR) was significantly reduced in the diabetes group (69.41 ± 31.13 mL/min/1.73 m^2^) compared to prediabetes subjects (91.84 ± 19.60 mL/min/1.73 m^2^; *p* = 0.010). Lipid profile analysis showed significantly lower total cholesterol (142.68 ± 32.72 mg/dL) and high-density lipoprotein (HDL) cholesterol (46.59 ± 13.01 mg/dL) in diabetic patients compared to prediabetic subjects (170.46 ± 34.15 mg/dL and 57.54 ± 15.61 mg/dL, respectively; *p* < 0.01 for both). Regarding anti-hyperglycemic medications in the diabetic group, 52.5% were treated with dipeptidyl peptidase-4 inhibitors (DPP-4), 38.3% with sulfonylureas (SU), 35.8% with alpha-glucosidase inhibitors (α-GI), 29.2% with insulin, 25.8% with thiazolidinediones (TZD), 14.2% with sodium-glucose cotransporter-2 inhibitors (SGLT2), and 0.8% with glucagon-like peptide-1 receptor agonists (GLP-1 RA).

### 2.2. Diabetes-Related Tongue Coating Metabolic Features

**Figure 1:**
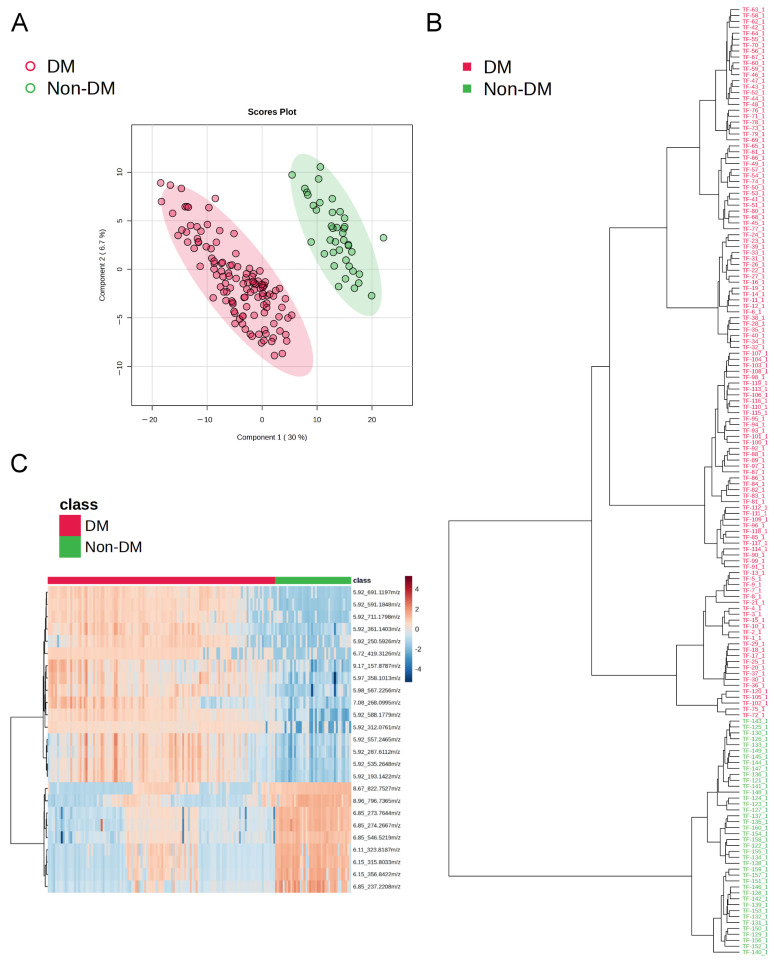
Analyses of inter-group differences in tongue coating metabolic features by non-diabetes and diabetes groups. (**A**) PLS-DA plot. (**B**) Phylogenetic tree plot. (**C**) Heatmap with top 25 differential metabolic features. DM: diabetes group; non-DM: non-diabetes group (normal + prediabetes).

**Figure 2:**
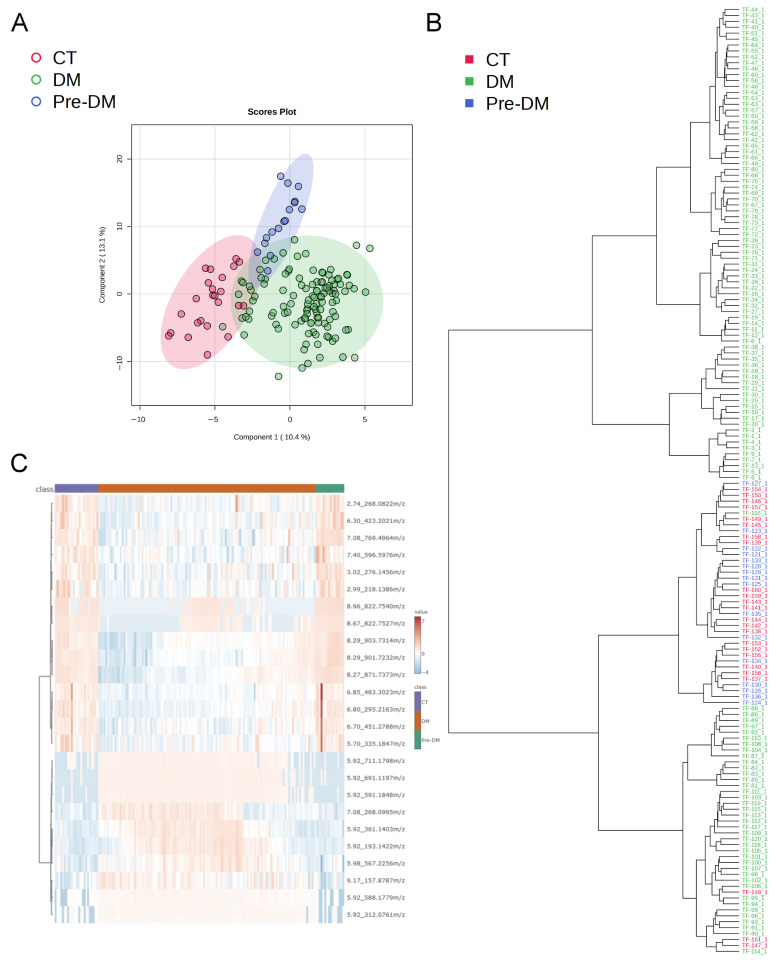
Analyses of inter-group differences in tongue coating metabolic features by normal control, prediabetes, and diabetes groups. (**A**) PLS-DA plot. (**B**) Phylogenetic tree plot. (**C**) Heatmap with top 25 differential metabolic features. CT: normal control group; DM: diabetes group; Pre-DM: prediabetes group.

Principal Component Analysis (PCA) was used to observe the variability and distribution among the control, prediabetes, and diabetes groups. The results indicated that most samples deviated within a reasonable range, with 15 out of 160 samples falling outside the Hotelling’s T^2^ threshold of 0.95 (Figure S1). Next, inter-group differences in tongue coating metabolite profiles were assessed using partial least squares discriminant analysis (PLS-DA), phylogenetic tree analysis, and heatmap clustering (Figure 1). From a total of 11,760 metabolic features, 312 features that exhibited more than a 2-fold difference, *p*-value < 0.05, and coefficient of variation < 100 between diabetes and non-diabetes (normal + prediabetes) groups were filtered and designated as diabetes-related metabolic features. The PLS-DA plot (Figure 1A) distinctly separated the T2DM group from the non-diabetes group, indicating unique metabolite signatures in diabetic subjects. This finding was further corroborated by the phylogenetic tree analysis (Figure 1B), which demonstrated clear metabolic clustering based on diabetes status. Moreover, the heatmap (Figure 1C) provided an additional layer of evidence for pronounced metabolic alterations linked to diabetes, revealing distinct group-specific clustering patterns. A comparison of metabolite compositions among the normal control, diabetes, and prediabetes groups also reveals observable inter-group differences (Figure 2). From a total of 11,760 metabolic features, 340 features that exhibited more than a 2-fold difference, *p*-value < 0.05, and coefficient of variation < 100 among normal control, prediabetes, and diabetes groups were filtered and designated as diabetes-related metabolic features. The clustering of the CT and Pre-DM groups suggests that the inter-group differences are not statistically significant (Figure 2A,B). The PLS-DA model constructed using DM and non-DM data is valid and demonstrates strong predictive power (Figure S2A). However, the model built with CT, Pre-DM, and DM group data, while performing perfectly on the training set, fails in prediction and is considered overfitted (Figure S2B). Nevertheless, these 340 features still yield significant clustering results when comparing each pair of groups (Figure S3) and conducting a small-scale PLS-DA analysis based on 340 features among CT, Pre-DM, and DM, performed on subjects aged > 40 years with a diabetes duration of <10 years (Figure S4).

### 2.3. Inter-Group Changes of Diabetes-Related Tongue Coating Metabolites

**Figure 3:**
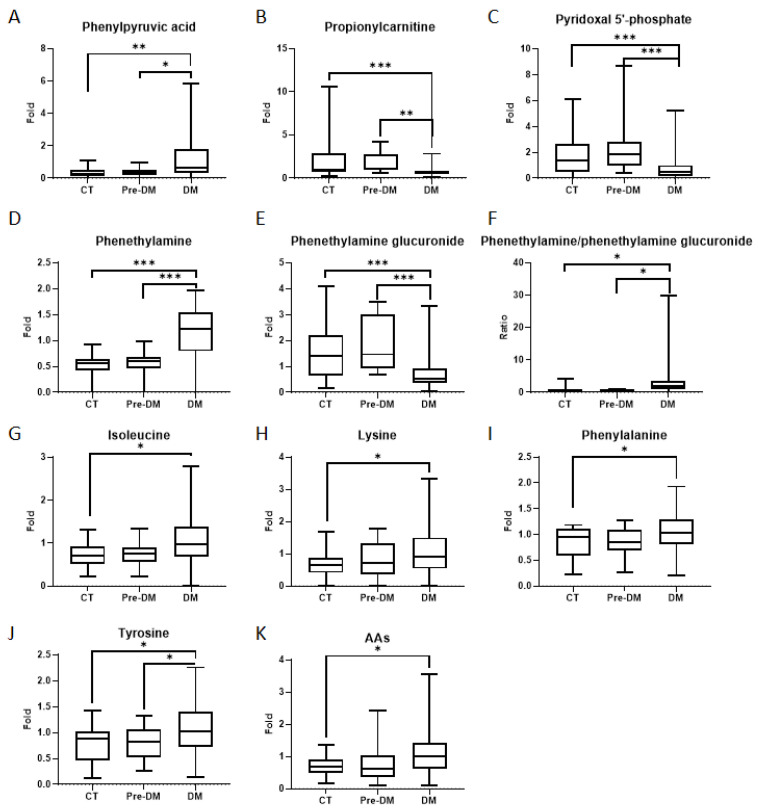
Changes in endogenous tongue coating metabolites related to diabetes. (**A**) Phenylpyruvic acid. (**B**) Propionylcarnitine. (**C**) Pyridoxal 5′-phosphate. (**D**) Phenethylamine. (**E**) Phenethylamine glucuronide. (**F**) Ratio of phenethylamine/phenethylamine glucuronide. (**G**) Isoleucine. (**H**) Lysine. (**I**) Phenylalanine. (**J**) Tyrosine. (**K**) Detected total amino acid fold change. CT: normal control group (*n* = 26); Pre-DM: prediabetes group (*n* = 14); DM: diabetes group (*n* = 120). * *p* < 0.05; ** *p* < 0.005; *** *p* < 0.0005.

Based on the differential metabolic features between the non-DM and DM groups, we further putatively annotated compound identities using the Human Metabolome Database (HMDB), leading to the discovery of potential differential metabolites between the two groups (Figure S5). We further selected metabolites previously reported to be associated with diabetes for analysis, and applied ANOVA together with correlation analysis to clarify the relationships between these metabolite targets, DM, and DM-related indicators in the tongue coating. Five putative endogenous metabolites were identified from diabetes-related tongue coating metabolic features (Figure 3, Table 2). Generally, the levels of phenylpyruvic acid (Figure 3A) and phenethylamine (Figure 3D) significantly increased in the diabetes group compared to the normal control and prediabetes groups. In contrast, the levels of propionylcarnitine (Figure 3B), pyridoxal 5′-phosphate (Figure 3C), and phenethylamine glucuronide (Figure 3E) significantly decreased in the diabetes group. The ratio of phenethylamine/phenethylamine glucuronide (Figure 3F) significantly increased in the diabetes group compared to the normal control and prediabetes groups.

### 2.4. Levels of Amino Acids in Tongue Coating Samples

Previous studies have revealed a link between amino acid metabolism and diabetes; therefore, we also analyzed the changes in amino acids detected in the mass spectrometry results [13,14,15,16]. The levels of 12 amino acids in tongue coating samples were measured by calculating the ratio of each target signal response to the mean response value. (Figure 3, Table 2). Specifically, the levels of isoleucine, lysine, phenylalanine, and tyrosine in the diabetes group were significantly higher compared to the normal control group (Figure 3G–K). Additionally, the level of tyrosine in the diabetes group was significantly higher compared to the prediabetes group (Figure 3J). Moreover, the detected total amino acid fold change in the diabetes group compared to the normal control group was significantly higher (Figure 3K).

### 2.5. Correlation of Tongue Coating Metabolites with Diabetes-Related Indices

**Figure 4:**
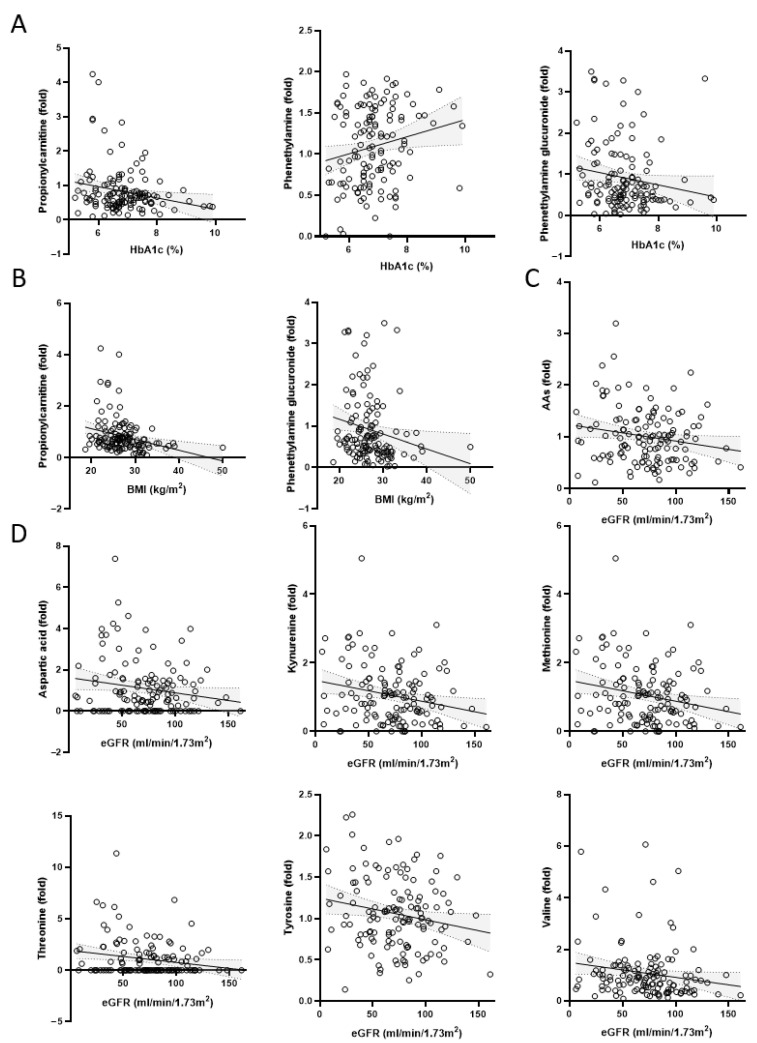
Correlation between tongue coating metabolite levels and diabetes-related indices. (**A**) HbA1c vs. propionylcarnitine, phenethylamine, or phenethylamine glucuronide. (**B**) BMI vs. propionylcarnitine or phenethylamine glucuronide. (**C**) eGFR vs. total detected amino acids. (**D**) eGFR vs. specific detected amino acid. (*n* = 130; from Diabetes + Prediabetes subjects).

Correlation analysis was conducted between the HbA1c, BMI, eGFR, and the corresponding levels of metabolites in tongue coating samples from prediabetes and diabetes groups. The results showed a negative correlation between HbA1c and the levels of propionylcarnitine (r = −0.2147; *p* = 0.0142), pyridoxal 5′-phosphate (r = −0.1185; *p* = 0.1792), and phenethylamine glucuronide (r = −0.1622; *p* = 0.0653), while a positive correlation was observed with phenylpyruvic acid (r = 0.155; *p* = 0.0783) and phenethylamine (r = 0.1918; *p* = 0.0288) levels. Notably, the correlations with propionylcarnitine and phenethylamine levels were significant (Figure 4A, Table 3). BMI showed a significant negative correlation with propionylcarnitine (r = −0.2753; *p* = 0.0015) and phenethylamine glucuronide (r = −0.1968; *p* = 0.0248) levels, while correlations with other metabolite levels showed no specific trends (Figure 4B, Table 4). The value of eGFR exhibited a significant negative correlation with the total detected amino acids (r = −0.237; *p* = 0.0066) level, including significant negative correlations with aspartic acid (r = −0.1746; *p* = 0.047), kynurenine (r = −0.2224; *p* = 0.011), methionine (r = −0.2378; *p* = 0.0065), threonine (r = −0.2002; *p* = 0.0224), tyrosine (r = −0.1881; *p* = 0.0321), and valine (r = −0.1724; *p* = 0.0499) levels (Figure 4C,D, Table 5).

### 2.6. Establishing a Diabetes Classification Index Using Tongue Coating Metabolomic Profiling Data

**Figure 5:**
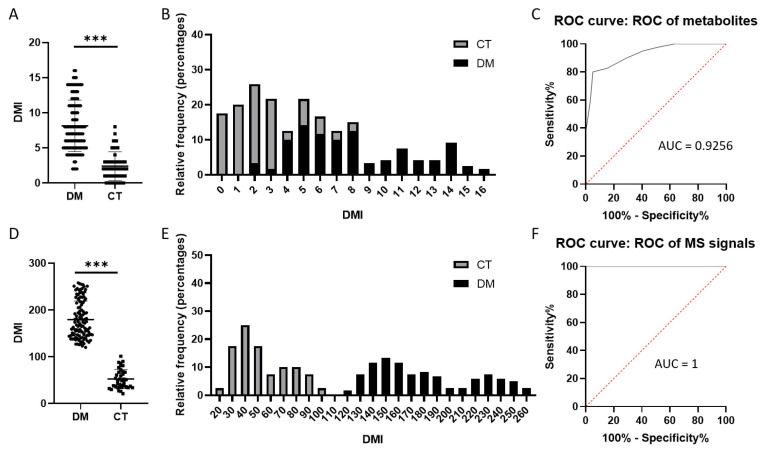
Establishment of DMI using tongue coating metabolic features as an indicator for distinguishing CT and DM. DMI was constructed based on identified candidate metabolic features, and (**A**) total DMI scores for subjects in the DM and non-DM groups, (**B**) proportional distribution across different DMI levels, and (**C**) ROC analysis results were summarized. Additionally, DMI was constructed using 312 metabolic features selected between the DM and non-DM groups, and (**D**) total DMI scores for subjects in the DM and non-DM groups, (**E**) proportional distribution across different DMI levels, and (**F**) ROC analysis results were summarized. *** *p* < 0.0001.

Candidate metabolites identified through differential analysis between the DM and non-DM groups were used to define a Diabetes Mellitus Index (DMI), based on the distribution of signal intensities across the two groups (Figure S5). While the highest mean MS signal was observed in the DM group, individual signals greater than the group mean were assigned DMI = 1, and those below the mean were assigned DMI = 0. Conversely, while the highest mean was found in the control (CT) group, signals above the mean were assigned DMI = 0, and those below were assigned DMI = 1. Using this definition, a total DMI score was calculated for each participant (Figure 5A). Distribution analysis revealed certain differences between the CT and DM groups, suggesting that this metabolite set has potential for DM classification (Figure 5B,C). Furthermore, when the same method was applied to 312 metabolic features selected based on inter-group differences, the total DMI differences among subjects became more pronounced (Figure 5D). Distribution analysis revealed a distinct separation point between the CT and DM groups, with a cutoff value of 110, indicating that this metabolite set provides stronger potential for accurate DM classification (Figure 5E,F). The discriminative power of these two DMI patterns is also corroborated by the clustering performance observed in the PLS-DA analysis (Figure S6).

## 3. Discussion

**Figure 6:**
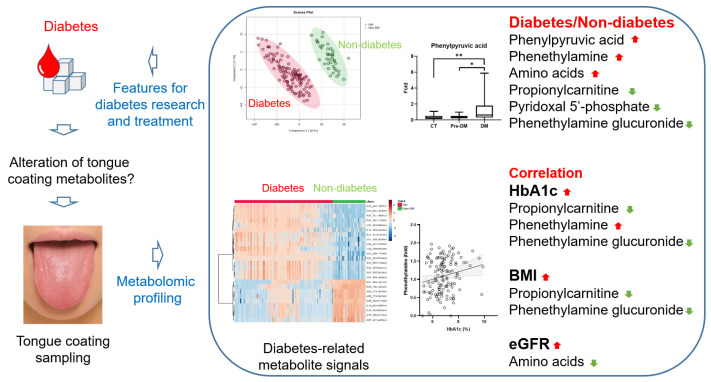
Application of tongue-coating metabolite profiling in diabetes research and treatment.

This study presents novel evidence supporting the feasibility of using tongue coating samples as a relatively non-invasive matrix for metabolic profiling in individuals with T2DM. Our findings demonstrate distinct alterations in specific endogenous metabolites and amino acids within the tongue coating of diabetic patients, which correlate with glycemic status, BMI, and renal function. These results suggest that tongue coating metabolomics reflects systemic metabolic dysregulation and could serve as a supplementary tool for monitoring metabolic status (Figure 6).

In the PLS-DA analysis, it can be observed that even under the supervised statistical model, the Pre-DM and CT groups largely cluster together (Figure 2B) and display similar heatmap distributions (Figure 2C). This pattern is further reflected when CT and Pre-DM are combined as the non-DM group, which shows a clear separation from the DM group (Figure 1). These results indicate that the tongue-coating metabolomic profiles of individuals with pre-diabetes do not differ substantially from those of healthy controls. Nevertheless, transitional metabolic changes can still be detected in the Pre-DM group, including metabolites such as phenethylamine, phenethylamine glucuronide, glutamic acid, isoleucine, lysine, phenylalanine, tyrosine, valine, and the overall amino acid pool (Figure 3 and Table 2). It is also noteworthy that in certain cases, the heatmap and phylogenetic distributions of the DM group appear closer to those of the CT and Pre-DM groups (non-DM group). This phenomenon may reflect the inherent metabolic heterogeneity during the development of diabetes and suggests that subtle metabolic phenotypes exist even within clinically defined groups. These findings highlight the potential and the challenges of using tongue-coating metabolomics for stratifying individuals across the diabetic spectrum (Figure 1 and Figure 2).

Phenylpyruvic acid, a metabolite derived from phenylalanine, was significantly elevated in the diabetic group. Previous studies have reported its accumulation in diabetic foot ulcers, where it contributes to impaired wound healing and chronic inflammation by activating the NLRP3 inflammasome [17]. Additionally, phenylpyruvic acid has been shown to inhibit glycolysis in hepatocytes [13], potentially contributing to glucose metabolic disturbances in diabetes. Our results suggest that increased phenylpyruvic acid in tongue coating is consistent with systemic metabolic disturbances and might reflect alterations in the oral microenvironment associated with T2DM (Figure 3A).

Propionylcarnitine, involved in fatty acid oxidation, was markedly reduced in diabetic subjects. L-carnitine and its derivatives are crucial for mitochondrial lipid metabolism and energy homeostasis [18]. Clinical trials have shown that propionylcarnitine supplementation can alleviate diabetic neuropathy and improve vascular function [18,19,20]. In our study, the reduced level of propionylcarnitine negatively correlated with both HbA1c and BMI, suggesting it may serve as a potential metabolic indicator reflecting glycemic control and obesity-related metabolic imbalance (Figure 3B and Figure 4A,B). These findings align with studies in African American women with T2DM, where reduced acylcarnitine levels were associated with impaired fatty acid β-oxidation and mitochondrial dysfunction [21].

Pyridoxal 5′-Phosphate (PLP), the active form of vitamin B6, plays essential roles in amino acid metabolism, neurotransmitter synthesis, and oxidative stress regulation. A growing body of evidence links reduced PLP levels with T2DM, especially in patients with early-stage nephropathy [22,23,24]. Our observation of decreased PLP levels in tongue coating aligns with these plasma-based findings, reflecting a potential association between T2DM and altered metabolic and oxidative status within the oral microenvironment (Figure 3C).

We observed elevated phenethylamine levels alongside a reduction in its conjugated form, phenethylamine glucuronide, in diabetic subjects. This resulted in a significantly increased phenethylamine/phenethylamine glucuronide ratio, which positively correlated with HbA1c and BMI (Figure 3D–F and Figure 4B). These findings suggest impaired glucuronidation, possibly due to reduced activity of UDP-glucuronosyltransferase (UGT) enzymes in diabetic states [25,26]. This altered ratio may provide insights into systemic or oral mucosal metabolic capacity under hyperglycemic stress and could serve as a potential indicator reflecting metabolic dysregulation.

Several amino acids, including isoleucine, lysine, phenylalanine, and tyrosine, were significantly elevated in the tongue coating of diabetic participants (Figure 3G–K). Prior research has shown that elevated levels of certain amino acids are predictive of T2DM onset and progression, likely due to their involvement in insulin resistance and altered protein metabolism [14,15]. Importantly, total amino acid content and several individual amino acids were negatively correlated with eGFR, consistent with the metabolic alterations observed in diabetic kidney dysfunction (Figure 4C,D). These findings suggest that tongue coating amino acid profiles may reflect systemic metabolic shifts associated with renal impairment in diabetes [16].

Metabolomics studies of diabetes have primarily focused on serum, plasma, urine, and saliva, which provide important information about systemic metabolic alterations [4,27,28]. Unlike these secreted body fluids, tongue coating samples additionally contain shed keratinized epithelial cells and microorganisms, and can be collected easily in a non-invasive manner. In our study, several putative metabolite trends identified in tongue coating samples were consistent with previous reports, further supporting the reproducibility of these metabolic changes during the progression of diabetes [13,14,15,16,17,18,19,20,21,22,23,24,25,26]. Moreover, establishing tongue-coating metabolomic analysis may facilitate its integration with microbiome profiling, thereby offering deeper insights into the interactions between environmental factors and host metabolism.

Establishing the DMI through MS metabolic features derived from tongue coating analysis appears to be a feasible exploratory approach for characterizing DM status. Moreover, our results indicate that increasing the number of MS features enhances the model’s discriminative power (Figure 5). This observation is consistent with the PLS-DA results obtained from metabolites and molecular features, indirectly highlighting the importance of the number of metabolic indicators in discriminative power (Figure S6). Therefore, without delving into molecular mechanisms, directly utilizing differences in MS metabolic features may be the most effective method for disease staging. This approach offers the advantage of requiring only minimal metabolomic profiling, such as using a single ESI+ mode, to provide reliable diagnostic criteria (Figure 5D–F). When constructing the multivariate model, the use of data from the DM and non-DM groups demonstrated stronger predictive power (Figure S2A). However, the use of data from the CT, Pre-DM, and DM groups performed poorly in the actual prediction (Figure S2B). Consequently, the DMI defined based on the DM and non-DM groups showed better discriminative ability (Figure 5). In addition, to ensure the generalizability of these criteria, fundamental limitations of mass spectrometry technology, including chromatographic conditions and instrument sensitivity-related biases, must still be addressed. In general, our proposed DMI remains in the experimental stage, serving primarily as a proof of concept. It currently lacks independent validation, and future work will need to establish appropriate model validation strategies to reconstruct the DMI and demonstrate whether it can provide additional value beyond existing clinical indicators.

Building on these promising findings, several avenues for future exploration emerge. First, the cross-sectional design provides a valuable snapshot of metabolic alterations associated with diabetes, laying the groundwork for future longitudinal and interventional studies to clarify causal relationships and assess the predictive or therapeutic potential of these putative biomarkers. Second, although all current analytical models consistently demonstrate the potential of tongue-coating metabolite profiling in diabetes identification (Figure 1, Figure 2 and Figure S4), expanding the sample sizes of the control and prediabetes groups in future studies could enhance generalizability and enable more nuanced subgroup analyses. Third, the use of positive ion mode in mass spectrometry offered a cost-effective strategy for detecting metabolite variations in tongue coating samples. Future research could benefit from incorporating broader sample preparation techniques, diverse chromatographic conditions, and complementary analytical modes to achieve a more comprehensive metabolic profile. Moreover, integrating variables such as oral hygiene, dietary habits, medication use, and microbial composition into study designs may help refine interpretations of metabolomic variability. Finally, while metabolite identities were matched using reference databases, targeted quantitative analyses and pathway enrichment studies will be instrumental in confirming these findings and uncovering their biological significance.

Although the metabolites observed in this study were associated with systemic conditions, it should be noted that the composition of tongue coating is strongly influenced by the oral microbiota and the local oral microenvironment. Consequently, some of the metabolic alterations identified here may originate from oral dysbiosis rather than solely reflecting systemic metabolic changes. This limitation highlights the need for future studies to integrate oral microbial profiling with metabolomic analyses to better disentangle local and systemic contributions.

## 4. Materials and Methods

### 4.1. Ethical Approval and Study Design

This is an observational, cross-sectional and clinical study. All the candidates underwent a standardized interview process. The study purpose, procedures, potential risks, and benefits were introduced to the participants. Subsequently, they signed an informed consent form. We conducted a cross-sectional, case-control, and observational study from 1 July 2019 to 31 December 2021. The trial was approved by the Institutional Review Board (IRB) of China Medical University Hospital (CMUH), Taichung, Taiwan (IRB reference number: CMUH109-REC1-138(AR-2), 25 September 2020) and that of Changhua Christian Hospital (CCH), Changhua, Taiwan (IRB reference number: CCH IRB 200414, 3 June 2020).

Diabetes mellitus (DM) was diagnosed in participants based on the American Diabetes Association (ADA) criteria, requiring either a fasting plasma glucose level ≥ 126 mg/dL or an HbA1c ≥ 6.5%, confirmed on two separate occasions. People with Pre-DM have a fasting glucose level of 100 to 125 mg/dL (impaired fasting glucose) or a 2 h plasma glucose level of 140 to 199 mg/dL after an oral glucose tolerance test (impaired glucose tolerance) on 2 separate occasions. Participants in the control group had a normal fasting glucose level (<100 mg/dL). Exclusion criteria were cancer, active liver disease, current pregnancy, active infection, and cerebrovascular disease.

All participants were recruited according to the clinical trial inspection regulations. After collecting tongue coating samples and preparing metabolite extracts, liquid chromatography–mass spectrometry (LC-MS) was conducted to acquire metabolite signal profiles. Subsequent data processing and analysis were conducted using relevant software.

### 4.2. Tongue Coating Sampling

A total of 160 tongue coating samples were collected, including 26 from CT subjects, 14 from Pre-DM subjects, and 120 from DM subjects. There was no strict requirement for subjects to fast before sampling. However, subjects were instructed to rinse their mouths with water three times, swallowing excess water. Using a tongue depressor (width 6 mm), tongue coating was scraped from the mid-tongue to the tip and collected in the centrifuge tube. The coating from both sides of the tongue was then collected in the same tube. The weight of tongue coating samples was recorded. Samples were temporarily stored in a 4 °C refrigerator or placed on ice. After each collection, the subject number, date, and other information were recorded. All batches of samples were centralized and stored at −80 °C.

### 4.3. Preparation of Tongue Coating Metabolite Samples

Tongue coating samples, added with ultrapure water in a ratio of 1 mg tongue coating to 10 μL water, were subjected to 10 min of ultrasonic vibration in icy water. After centrifugation at 14,000× *g* for 10 min, 80 μL of the supernatant was mixed with 320 μL of 100% methanol, placed at −80 °C for 3 h, and then brought to room temperature. After centrifugation at 14,000× *g* for 10 min, 320 μL of the supernatant was vacuum-dried, and the dried samples were stored at −20 °C. Before analysis, samples were reconstituted with 60 μL ultrapure water, centrifuged at 14,000× *g* for 10 min, and the supernatant was transferred to sample vials for LC-ESI-MS analysis. The quality control (QC) sample was prepared by pooling 5 μL from each individual sample.

### 4.4. LC-ESI-MS Analysis

The analytical setup employed a high-resolution LC-ESI-MS platform, integrating a Waters ACQUITY UPLC I-Class system with a VION quadrupole time-of-flight (TOF) mass spectrometer equipped with an ESI/APCI ionization source (Waters, Milford, MA, USA). Chromatographic separation was carried out at a flow rate of 0.2 mL/min and a column temperature of 35 °C. A BEH C18 column (2.1 × 100 mm, Waters, Milford, MA, USA) was used for reversed-phase liquid chromatography (RPLC), with a sample injection volume of 5 μL. The gradient elution began with 99% of mobile phase A (ultrapure water containing 0.1% formic acid) and 1% of mobile phase B (methanol with 0.1% formic acid). This composition was maintained for 0.5 min, then gradually increased to 90% B over 5.5 min, held at that level for 1 min, and subsequently returned to 1% B within 1 min. The column was re-equilibrated by flushing with 1% B for an additional 4 min. Mass spectrometric data were collected in positive ion mode (ESI+) under the following operating parameters: capillary voltage set at 2.5 kV, source temperature at 100 °C, desolvation temperature at 250 °C, cone gas flow at 10 L/h, and desolvation gas flow at 600 L/h. Data acquisition was performed in MS^E^ mode, scanning a mass-to-charge (*m*/*z*) range of 100–1000 with a scan duration of 0.5 s. The resulting chromatograms were processed using Waters UNIFI 1.8.2 software, which provided visual outputs and integrated signal areas for analysis.

### 4.5. Data Processing

The analytical data from nine interval QC sample injections were processed using Progenesis QI v3.0 for peak detection and alignment, and signal normalization, followed by PCA analysis to confirm the clustering of QC samples. Signals extracted from chromatography-mass spectrometry were analyzed for inter-group differences using Progenesis QI and MetaboAnalyst 6.0. The median CV for 312 features was 10.49%, and 67.3% of features showed an intra-batch CV < 20%. Inter-Group signals meeting the criteria of >2-fold difference, *p*-value < 0.05, and coefficient of variation < 100 were subjected to further analysis. The samples near the edge of Hotelling’s T^2^ ellipse were retained in the final analysis as their clinical characteristics were consistent with the group, and they did not disproportionately influence the PCA model. All sample features were retained, and any remaining missing values were set to zero. Partial least squares discriminant analysis (PLS-DA), phylogenetic tree analysis, and heatmap analysis were employed to identify inter-group signal differences between the control and diabetes groups. The identity of the metabolite was putatively annotated according to the Metabolomics Standards Initiative (MSI) Level 2. Potential metabolite identities associated with inter-group differential signals were matched in the Human Metabolome Database (HMDB). High-resolution mass spectrometry signals were matched to metabolites based on the [M + H]^+^ *m*/*z* values (molecular weight tolerance ± 5 ppm), and metabolite identification was further confirmed by referencing the Kyoto Encyclopedia of Genes and Genomes (KEGG) database together with the predicted retention times.

### 4.6. Statistical Analysis

To satisfy the assumptions of parametric testing, all metabolic features (*m*/*z* intensities) were log-transformed (log10) and auto-scaled. Normality was confirmed using the Shapiro–Wilk test prior to performing correlation analyses and ANOVA. A Response Screening Analysis was employed to evaluate 2040 features while controlling for the False Discovery Rate (FDR). This framework allowed us to account for demographic confounders by including age, sex, and medication as covariates, ensuring that the metabolic differences between the unmatched healthy control group and the age-matched progression groups (Pre-DM and DM) were driven by disease pathology. Preliminary analyses examined the effects of age, medication status, and sample size imbalance across groups. Since ANCOVA indicated that these covariates had no significant predictive power for the dependent variable and did not affect the main findings, they were not included in the final adjusted model.

Statistical analysis, including mean, standard deviation, ANOVA, correlation analysis, and plotting, was performed using MetaboAnalyst 6.0 and GraphPad Prism software version 8.0.1 (GraphPad Software, San Diego, CA, USA). Statistical comparisons were analyzed using GraphPad Prism software with one-way ANOVA and multicomparison tests, as well as unpaired *t*-tests. Significance was considered at a probability error (*p*) < 0.05, and all *p*-values were two-tailed. The plots were created using means with standard deviation for error bars.

Diabetes Mellitus Index (DMI): After filtering metabolites and molecular features with inter-group differences from the DM and non-DM groups, the individual mass spectrometry signal intensity is denoted as *I*, and the average intensity as *M*. If the maximum fold change occurs in the DM group and *I* > *M*, then DMI = 1. Conversely, if the maximum fold change occurs in the non-DM group and *I* < *M*, then DMI = 1.

## 5. Conclusions

This study demonstrates that tongue coating metabolite profiling can be used to distinguish metabolic profiles among individuals with normal glycemic status, prediabetes, and T2DM. Specific metabolite alterations, including elevated phenylpyruvic acid and phenethylamine, and reduced propionylcarnitine, pyridoxal 5′-phosphate, and phenethylamine glucuronide, were associated with clinical indices such as HbA1c, BMI, and eGFR. These findings suggest that the metabolic composition of tongue coating not only reflects systemic metabolic disturbances but also holds potential as a non-invasive diagnostic and prognostic tool for diabetes. By identifying potential biomarkers linked to protein glycation, lipid dysregulation, vitamin B6 metabolism, and renal function, this study offers insights into the pathophysiological mechanisms of diabetes. The accessibility and simplicity of tongue coating sampling further support its clinical applicability in community screening or outpatient monitoring. To fully realize its clinical utility, future research should focus on longitudinal validation, pathway-based interpretation, and integration with microbial profiling.

## Figures and Tables

**Table 1:** Clinical and biochemical characteristics of participants.

	Control Group (*n* = 26)	Pre-DM (*n* = 14)	Type 2 DM (*n* = 120)	*p* Value
Age (y/o)	53.96 ± 21.42	64.57 ± 9.17	64.89 ± 9.78	0.000
Gender (M/F)	13/13	3/11	59/61	0.136
BMI (kg/m^2^)	24.09 ± 3.76	24.43 ± 2.58	27.53 ± 4.71	0.000
Ac sugar (mg/dL)	-	111.00 ± 13.21	133.95 ± 36.60	0.022
HbA1c (%)	-	5.88 ± 0.42	6.96 ± 0.85	0.000
Cr (mg/dL)	-	0.71 ± 0.11	1.28 ± 1.17	0.071
eGFR (ml/min/1.73 m^2^)	-	91.84 ± 19.60	69.41 ± 31.13	0.010
GOT (mg/dL)	-	21.40 ± 7.41	26.20 ± 19.05	0.432
GPT (mg/dL)	-	21.36 ± 9.90	24.78 ± 14.71	0.398
TC (mg/dL)	-	170.46 ± 34.15	142.68 ± 32.72	0.004
TG (mg/dL)	-	109.85 ± 48.39	129.90 ± 73.56	0.339
HDL (mg/dL)	-	57.54 ± 15.61	46.59 ± 13.01	0.005
LDL (mg/dL)	-	92.26 ± 26.63	77.13 ± 23.01	0.024
HOMA-IR	-	0.59 ± 0.73	0.89 ± 1.85	0.540
Anti-Hyperglycemic Agents				
Insulin	-	-	35 (29.2%)	-
SU	-	-	46 (38.3%)	-
TZD	-	-	31 (25.8%)	-
a-GI	-	-	43 (35.8%)	-
DPP-4	-	-	63 (52.5%)	-
SGLT2	-	-	17 (14.2%)	-
GLP-1 RA	-	-	1 (0.8%)	-

BMI (body mass index), HOMA-IR (Homeostatic model assessment for insulin resistance). Anti-Hyperglycemic Agents: SU (sulfonylureas), TZD (thiazolidinediones), a-GI (alpha-glucosidase inhibitors), DPP-4 (dipeptidyl peptidase-4 inhibitors), SGLT2 (sodium-glucose cotransporter 2 inhibitors), and GLP-1 RA (glucagon-like peptide-1 receptor agonists). *p*-values were performed by Chi-square test (or Fisher’s exact) for categorical variables and ANOVA for continuous variables.

**Table 2:** ANOVA analysis of inter-group metabolite levels between normal control, prediabetes, and diabetes groups.

Metabolites	CT vs. Pre-DM	CT vs. DM	Pre-DM vs. DM	F	*p* Value
Phenylpyruvic acid	0.009301	−0.8550	−0.8643	8.546	0.0003
Pyridoxal 5′-phosphate	−0.2474	0.7328	0.9803	23.09	<0.0001
Propionylcarnitine	−0.4912	1.126	1.617	20.14	<0.0001
Phenethylamine	0.2796	1.326	1.046	33.97	<0.0001
Phenethylamine glucuronide	−0.01120	−0.6225	−0.6113	17.48	<0.0001
Phenethylamine/phenethylamine glucuronide	0.3650	−2.446	−2.811	6.316	0.0023
Aspartic acid	0.08286	−0.5774	−0.6603	3.886	0.0225
Glutamic acid	−0.07963	−0.3881	−0.3085	1.5	0.2263
Histidine	−0.9533	−0.4983	0.4550	1.675	0.1907
Isoleucine	−0.01420	−0.3156	−0.3014	5.834	0.0036
Kynurenine	0.02277	−0.2908	−0.3135	2.087	0.1275
Lysine	−0.1320	−0.3988	−0.2668	4.07	0.0189
Methionine	0.3652	−0.05872	−0.4240	1.391	0.2518
Phenylalanine	−0.03460	−0.2215	−0.1869	5.446	0.0052
Threonine	0.2932	−0.5555	−0.8487	2.676	0.072
Tryptophan	0.09977	−0.1863	−0.2860	2.682	0.0716
Tyrosine	−0.01528	−0.2840	−0.2687	6.674	0.0017
Valine	−0.1008	−0.4812	−0.3804	3.176	0.0444
AAs (detected total amino acid fold change)	−0.03890	−0.3554	−0.3165	5.224	0.026

**Table 3:** Correlation analysis between HbA1c and tongue coating metabolite levels.

Correlation to HbA1c	r	*p* Value	R^2^
Phenylpyruvic acid	0.155	0.0783	
pyridoxal 5′-phosphate	−0.1185	0.1792	
Propionylcarnitine	−0.2147	0.0142	0.04608
Phenethylamine	0.1918	0.0288	0.03680
Phenethylamine glucuronide	−0.1622	0.0653	0.02630
Aspartic acid	0.1069	0.2261	
Glutamic acid	0.06149	0.4871	
Histidine	0.04194	0.6356	
Isoleucine	0.1406	0.1105	
Kynurenine	0.02656	0.7642	
Lysine	0.04665	0.5981	
Methionine	0.008687	0.9219	
Phenylalanine	0.1612	0.0669	
Threonine	0.08571	0.3322	
Tryptophan	0.005558	0.95	
Tyrosine	0.03571	0.6867	
Valine	0.01748	0.8435	
AAs (detected total amino acid fold change)	0.08732	0.3232	

**Table 4:** Correlation analysis between BMI and tongue coating metabolite levels.

Correlation to BMI	r	*p* Value	R^2^
Phenylpyruvic acid	−0.1025	0.246	
Propionylcarnitine	−0.2753	0.0015	0.07581
pyridoxal 5′-phosphate	−0.1708	0.052	
Phenethylamine	−0.0106	0.9037	
Phenethylamine glucuronide	−0.1968	0.0248	0.03872
Aspartic acid	−0.02975	0.7369	
Glutamic acid	0.03094	0.7268	
Histidine	0.09845	0.2651	
Isoleucine	0.05222	0.5551	
Kynurenine	0.01249	0.8878	
Lysine	0.1264	0.1519	
Methionine	−0.03091	0.727	
Phenylalanine	0.04832	0.5851	
Threonine	−0.08513	0.3356	
Tryptophan	−0.1082	0.2203	
Tyrosine	0.1159	0.189	
Valine	−0.01265	0.8864	
AAs (detected total amino acid fold change)	0.005292	0.9523	

**Table 5:** Correlation analysis between eGFR and tongue coating metabolite levels.

Correlation to eGFR	r	*p* Value	R^2^
Phenylpyruvic acid	0.1011	0.2525	
Propionylcarnitine	0.09955	0.2598	
pyridoxal 5′-phosphate	0.0241	0.7855	
Phenethylamine	0.01478	0.867	
Phenethylamine glucuronide	0.01123	0.8991	
Aspartic acid	−0.1746	0.047	0.03047
Glutamic acid	−0.1662	0.0588	
Histidine	−0.03653	0.6799	
Isoleucine	−0.1434	0.1036	
Kynurenine	−0.2224	0.011	0.04948
Lysine	−0.09013	0.3078	
Methionine	−0.2378	0.0065	0.04948
Phenylalanine	−0.1239	0.1602	
Threonine	−0.2002	0.0224	0.04008
Tryptophan	−0.09298	0.2927	
Tyrosine	−0.1881	0.0321	0.03537
Valine	−0.1724	0.0499	0.02971
AAs (detected total amino acid fold change)	−0.237	0.0066	0.03282

## Data Availability

The original contributions presented in this study are included in the article/Supplementary Materials. Further inquiries can be directed to the corresponding authors.

## Supplementary Materials

The following supporting information can be downloaded at: https://www.mdpi.com/article/10.3390/ijms27083375/s1.

## Abbreviations

ADA: American Diabetes Association; ANOVA: Analysis of Variance; APCI: Atmospheric Pressure Chemical Ionization; BMI: Body Mass Index; DPP-4: Dipeptidyl Peptidase-4; eGFR: Estimated Glomerular Filtration Rate; ESI: Electrospray Ionization; GLP-1 RA: Glucagon-like Peptide-1 Receptor Agonist; HbA1c: Hemoglobin A1c; HDL: High-Density Lipoprotein; HMDB: Human Metabolome Database; IRB: Institutional Review Board; LC-MS: Liquid Chromatography–Mass Spectrometry; LC-ESI-MS: Liquid Chromatography–Electrospray Ionization–Mass Spectrometry; PLP: Pyridoxal 5′-Phosphate; PLS-DA: Partial Least Squares Discriminant Analysis; RPLC: Reversed-Phase Liquid Chromatography; SGLT2: Sodium-Glucose Cotransporter-2; TOF: Time-of-Flight; T2DM: Type 2 Diabetes Mellitus; TZD: Thiazolidinedione; UGT: UDP-glucuronosyltransferase; UPLC: Ultra-Performance Liquid Chromatography.
