# Supplementary material for: Metabolomic Profiling of Tongue Coating Reveals Potential Molecular Features Linked to Type 2 Diabetes Progression
Source: Int J Mol Sci. 2026 Apr 9;27(8):3375. doi: 10.3390/ijms27083375 (PMC13116826; doi:10.3390/ijms27083375)
Supplement: Supplementary file 1 [file ijms-27-03375-s001.zip › ijms-4197098-supplementary.pdf]

Supplementary Figures

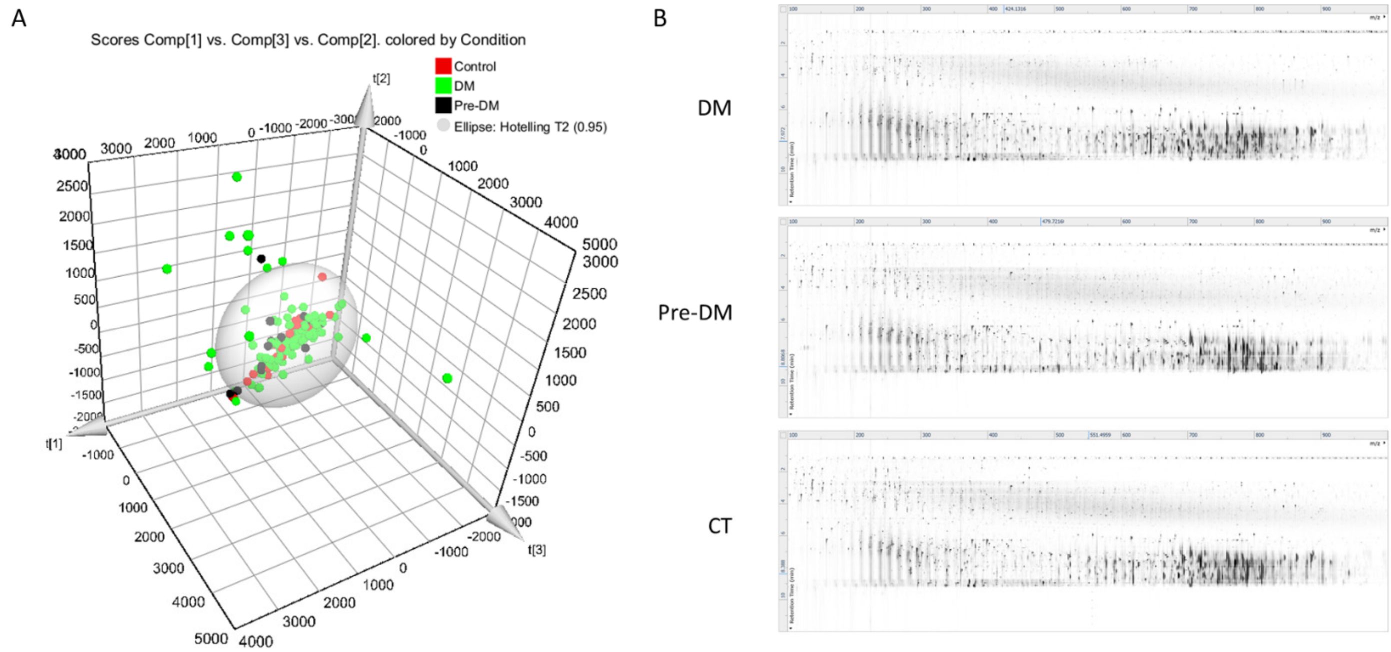

Figure S1. Analyses of inter-group differences in tongue coating metabolite signals. (A) A total of 11,760 mass spectrometry signals was subjected Principal Component Analysis (PCA). (B) Representative 2-D profiles of LC-MS for tongue coatings. DM: diabetes group; Pre-DM: prediabetes group; CT: control group.

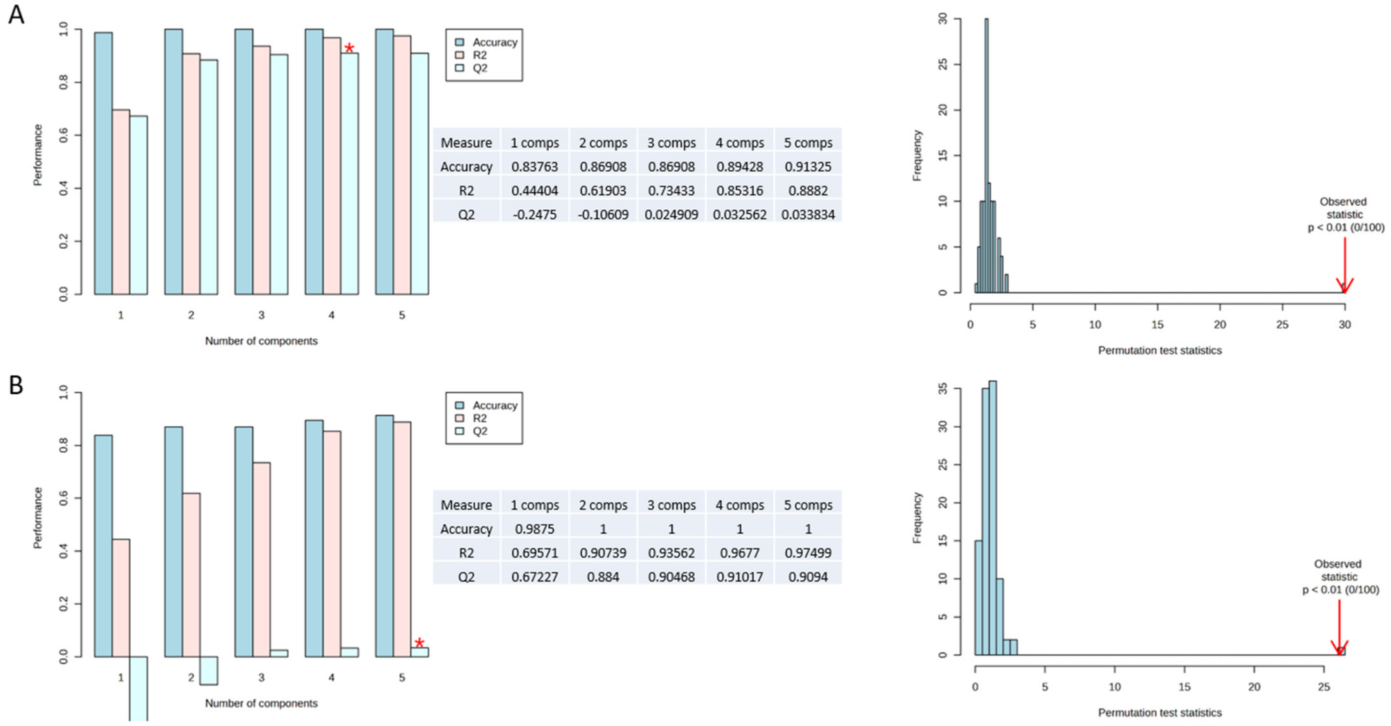

Measure	1 comps	2 comps	3 comps	4 comps	5 comps
Accuracy	0.83763	0.86908	0.86908	0.89428	0.91325
R2	0.44404	0.61903	0.73433	0.85316	0.8882
Q2	-0.2475	-0.10609	0.024909	0.032562	0.033834

Measure	1 comps	2 comps	3 comps	4 comps	5 comps
Accuracy	0.9875	1	1	1	1
R2	0.69571	0.90739	0.93562	0.9677	0.97499
Q2	0.67227	0.884	0.90468	0.91017	0.9094

Figure S2. Cross-validation and permutation test of the PLS-DA model: (A) DM versus non-DM. (B) CT, Pre-DM, and DM.

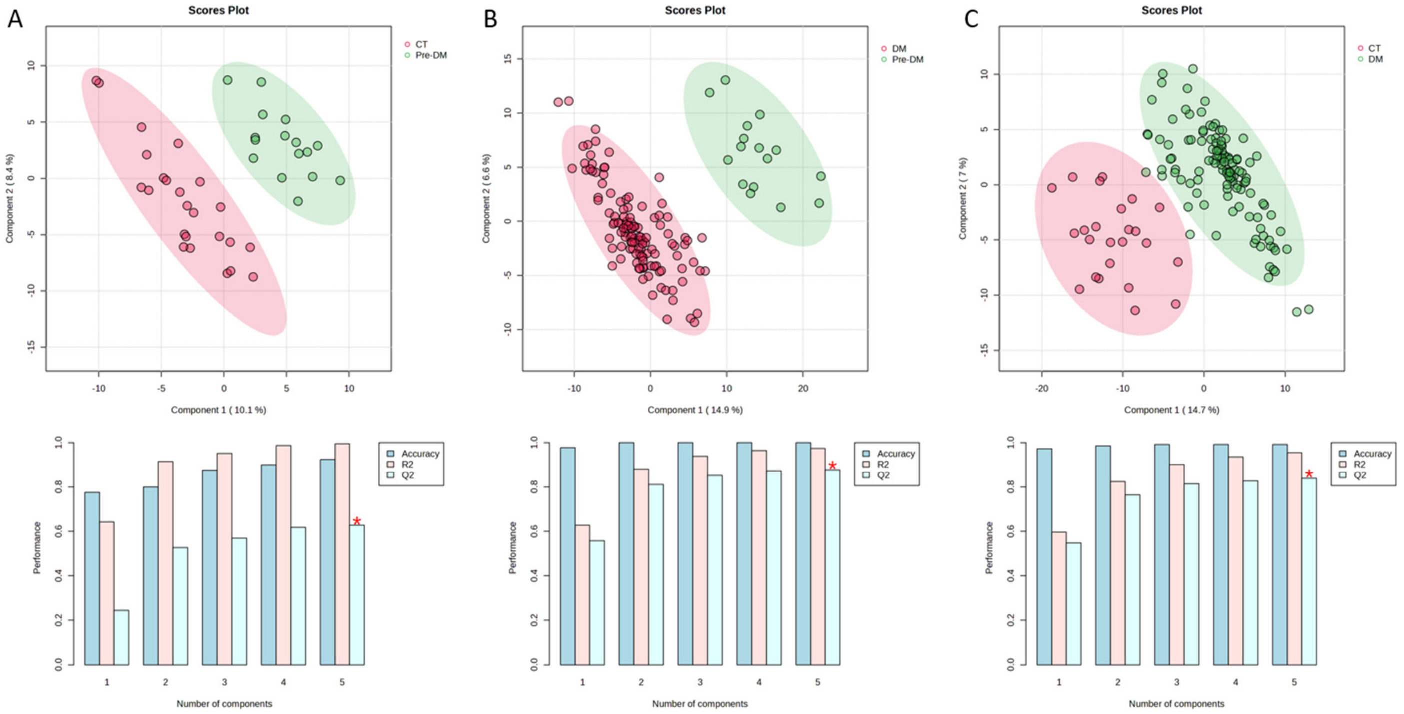

Figure S3. PLS-DA pairwise group comparisons based on 340 features among CT, Pre-DM, and DM. (A) CT versus Pre-DM, (B) Pre-DM versus DM, and (C) CT versus DM.

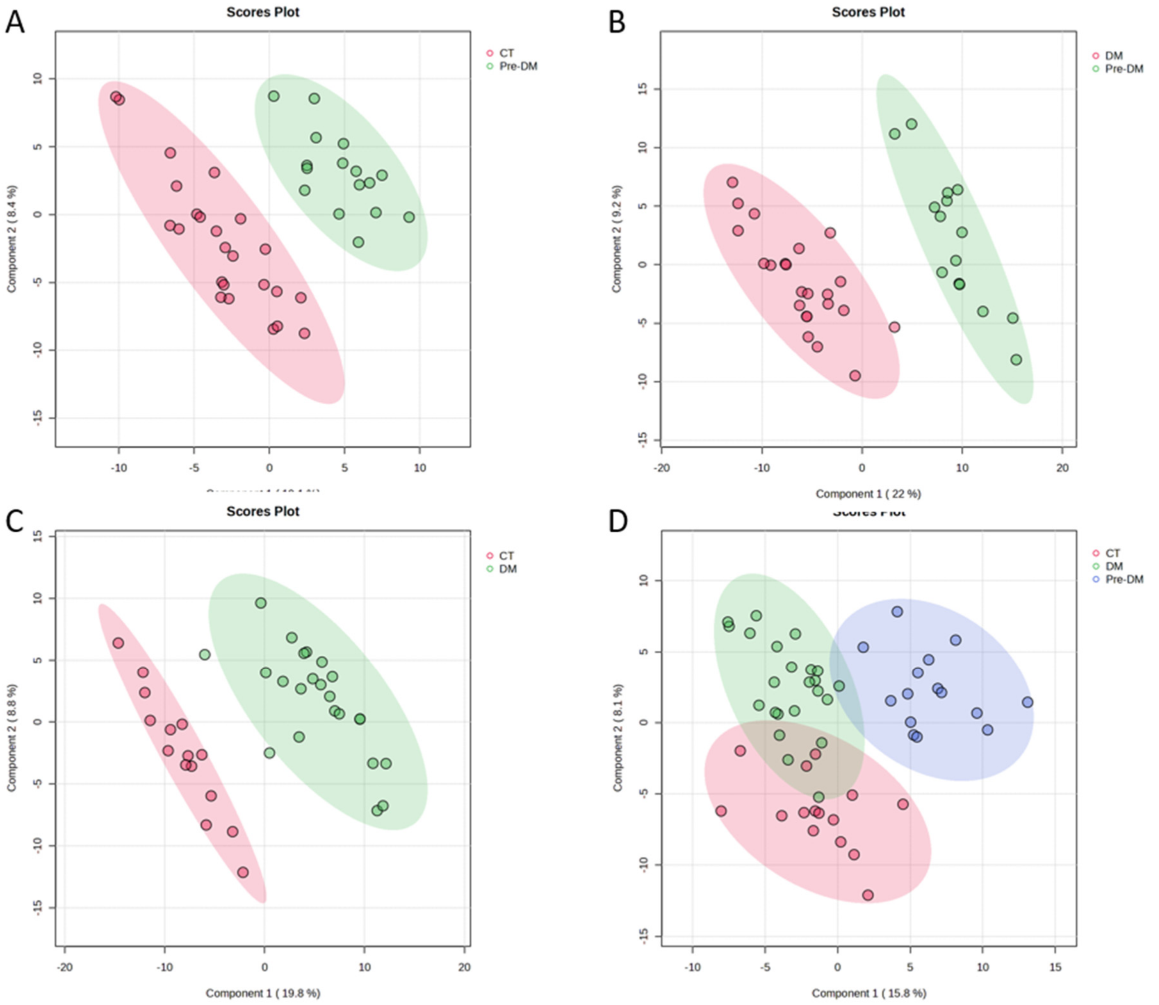

Figure S4. Small-scale PLS-DA analysis based on 340 features among CT, Pre-DM, and DM, performed on subjects aged >40 years with a diabetes duration <10 years. (A) CT versus Pre-DM, (B) CT versus Pre-DM, (C) CT versus DM, and (D) CT versus Pre-DM versus DM.

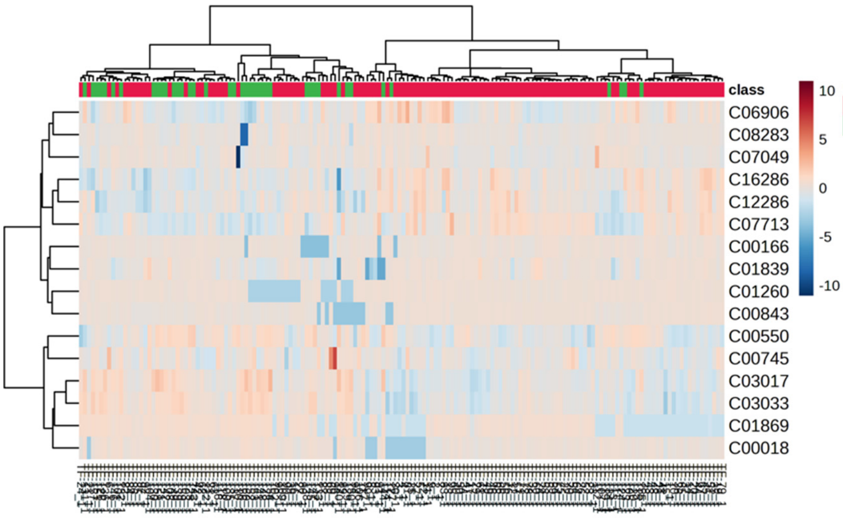

KEGG	Name	DM/non-DM	p value	FDR
C00843	(-)-Menthone	7.20	3.4E-07	3.4E-07
C08283	(S)-Homostachydrine	2.86	9.2E-08	9.2E-08
C12286	alpha-Ionone	2.32	2.2E-06	2.2E-06
C06906	Chlorpromazine	2.62	1.7E-04	1.7E-04
C01260	Diadenosine tetraphosphate	2.14	6.2E-03	6.2E-03
C01869	D-synephrine	0.48	3.9E-04	3.9E-04
C01839	Ethyl benzoate	7.40	2.9E-02	2.9E-02
C16286	Geosmin	2.31	1.3E-06	1.3E-06
C07049	Imipramine	2.40	1.9E-01	1.9E-01
C00745	Nicotine	3.19	3.0E-01	3.0E-01
C07713	Nitrendipine	4.09	4.2E-13	4.2E-13
C03033	3-Methoxy-4-hydroxyphenylglycol glucuronide	0.49	3.0E-08	3.0E-08
C00166	Phenylpyruvic acid	3.41	5.5E-05	5.5E-05
C03017	Propionylcarnitine	0.36	2.8E-09	2.8E-09
C00018	Pyridoxal 5'-phosphate	0.34	5.0E-10	5.0E-10
C00550	2beta-Hydroxytestosterone	0.50	1.9E-04	1.9E-04

Figure S5. Hierarchical Clustering Heatmaps of identified differential metabolites between DM and non-DM. DM: diabetes group; Non-DM: non-diabetes group (normal + prediabetes).

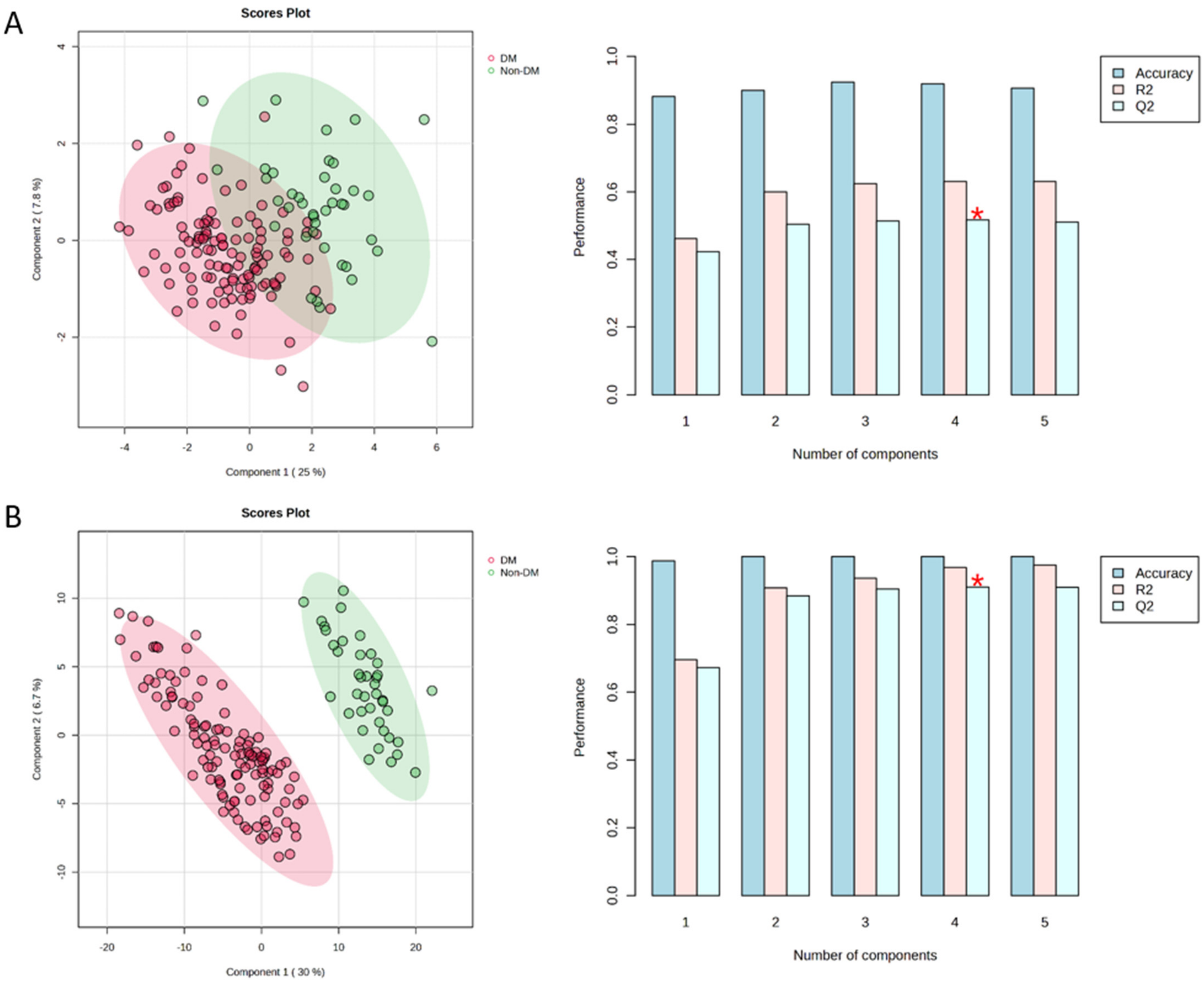

Figure S6. PLS-DA analyses based on (A) 16 identified metabolites and (B) 312 molecular features.
